# Editorial for Gels 6th Anniversary Special Issue

**DOI:** 10.3390/gels8050249

**Published:** 2022-04-19

**Authors:** Esmaiel Jabbari, Gulden Camci-Unal

**Affiliations:** 1 Biomaterials and Tissue Engineering Laboratory, Department of Chemical Engineering, University of South Carolina, Columbia, SC 29208, USA; 2 Department of Chemical Engineering, University of Massachusetts Lowell, One University Avenue, Lowell, MA 01854, USA; gulden_camciunal@uml.edu

This Special Issue celebrates many outstanding quality papers published in *Gels* over the past six years since its first issue was published in 2015. The discovery and development of novel gels are vital to solving many global challenges from sustainable, eco-friendly, regenerative, and minimally invasive materials to materials for renewable energy, soil preservation, sustainable food production, clean water, and preventing the spread of infectious diseases during pandemics. The papers in this Special Issue showcase new developments in gels and their application in drug delivery, regenerative medicine, wound healing, cultivated meat, xerogels, oleogels, gels for radiation dosimetry, and environmentally responsive gels. Xerogels are gels that retain their porous structure after drying at ambient condition. In this regard, Prostredny et al. [1] investigate the impact of replacing resorcinol in resorcinol–formaldehyde gels with s-triazine precursors on the crosslinking and texture of the resulting xerogels. The substitution of the z-triazine precursor ranging from tri-hydroxyl to tri-amine allows changing the chemistry and acid–base properties of the gelation reaction. In a related work, Martin et al. [2] simulate the impact of synthetic parameters on textural and fractal properties of resorcinol–formaldehyde organic gels by a 3D-lattice-based Monte Carlo simulation approach. In another related work, Berg et al. [3] report the swelling and thermal sensitivity of novel cryo-clay-silica gels based on N-isopropylacrylamide and compare the results with those of clay-, silica-clay, and cryo-clay gels. The classical parabolic models are unable to predict non-Fickian sorption kinetics in swelling glass–gel drug delivery systems. In this regard, Adrover et al. [4] extend the stochastic Poisson–Kac model to describe the temporal evolution of glass–gel and gel–solvent interfaces and predict the effect of gel relaxation time on sorption and drug release kinetics. New treatment methods are needed to improve the quality-of-life of patients with chronic wounds. In this regard, Firlar et al. [5] review the advantages of multifunctional engineered hydrogel dressings over traditional methods to treat chronic wounds at different stages of healing. Conventional microcarriers used for the expansion of stem cells require the detachment and separation of the cells from the carrier prior to use in the clinic for tissue regeneration. Jabbari and Sepahvandi [6] describe a novel approach to use fetal or adult animal tissues as microcarriers for the expansion and implantation of stem cells without the need to detach the expanded cells from the carrier. In this approach, fetal or adult animal tissue is minced, decellularized, freeze-dried, ground, and sieved to produce microgels. Then, stem cells are expanded on the microgels, and the cell-expanded microgels are suspended in an alginate gel and injected into a tissue cavity for regeneration without the need for cell separation. Cultivated meat, which is meat cultured directly from animal cells, could eliminate the need to raise animals, which could potentially reduce land and water use, greenhouse gases, and pollution. Furthermore, cultivated lean meat could usher in healthier lifestyles and lower the incidence of heart disease in world population. In this regard, Martins et al. [7] investigate the impact of replacing animal fat with edible oleogels based on beeswax on hardness, adhesivity, and omega-3 availability in meat-based spreadable products and their processing. In a related work, Barroso et al. [8] investigate the effect of combining gelling agents such as berry and sunflower wax with glycerol monostearate on the structural formation and gelation of flaxseed oil to generate nutritional gels with tailored physiochemical and mechanical properties. Nanostructured gels are used in cancer therapy to target antibodies, cytokines, proteins, DNAs, and RNAs to cells in the tumor. Ghaeini-Hesaroeiye et al. [9] review chemical moieties that impart thermosensitivity to nanogels with tunable responses for targeted tumor delivery in cancer patients. In a related work, Pinelli et al. [10] review surface functionalization and coating strategies for the selective modification of nanoparticles and nanogels and highlight their application in targeted drug delivery. New sorbents are needed to increase the recovery of toxic acidic ores from leachate for environmental safety. In this regard, Hamza et al. [11] investigate the material properties, sorption isotherms, and uptake kinetics of leachate from algal/polyethyleneimine composite hydrogels synthesized by the quaternization of the algal support with glycidyltrimethylammonium chloride. In a work related to radiation dosimetry, Marrale and d’Errico [12] review the physical and chemical properties of tissue-equivalent Fricke and polyacrylamide gels for application in 3D ionizing radiation dosimetry to protect the patient from radiation and ensure the high quality of radiation treatment. The papers in this Special Issue highlight the enormous application of gels in medicine, pharmaceutics, cancer treatment, food production, radiation monitoring, and environmental protection to improve the quality-of-life of the world population.
